# Extensive Metabolic Profiles of Leaves and Stems from the Medicinal Plant *Dendrobium* *officinale* Kimura et Migo

**DOI:** 10.3390/metabo9100215

**Published:** 2019-10-04

**Authors:** Hua Cao, Yulu Ji, Shenchong Li, Lin Lu, Min Tian, Wei Yang, Han Li

**Affiliations:** 1 Institute of Flower Research, Yunnan Academy of Agricultural Sciences, No. 2238, Beijing Road, Kunming 650200, Yunnan, China; caohua19811227@126.com (H.C.); kmlijinbin@163.com (L.L.); tminfl@yeah.net (M.T.); cygsgg141111@163.com (W.Y.); 2 National Engineering Technology Research Center for Ornamental Horticulture, No. 2238, Beijing Road, Kunming 650200, Yunnan, China; 3 College of Landscape and Horticulture, Yunnan Agricultural University, No. 452, Fengyuan Road, Kunming 650201, Yunnan, China; July55775@outlook.com

**Keywords:** *Dendrobium officinale*, widely targeted metabolomics, bioactive compounds, plant organs

## Abstract

*Dendrobium officinale* Kimura et Migo is a commercially and pharmacologically highly prized species widely used in Western Asian countries. In contrast to the extensive genomic and transcriptomic resources generated in this medicinal species, detailed metabolomic data are still missing. Herein, using the widely targeted metabolomics approach, we detect 649 diverse metabolites in leaf and stem samples of *D. officinale*. The majority of these metabolites were organic acids, amino acids and their derivatives, nucleotides and their derivatives, and flavones. Though both organs contain similar metabolites, the metabolite profiles were quantitatively different. Stems, the organs preferentially exploited for herbal medicine, contained larger concentrations of many more metabolites than leaves. However, leaves contained higher levels of polyphenols and lipids. Overall, this study reports extensive metabolic data from leaves and stems of *D. officinale*, providing useful information that supports ongoing genomic research and discovery of bioactive compounds.

## 1. Introduction

The genus *Dendrobium* is one of the largest genera of the Orchidaceae comprising 1500–2000 species widely distributed in Asia and Northern Australia [1]. Among these species, *Dendrobium officinale* Kimura et Migo is a commercially and pharmacologically valuable species. *D. officinale* has been long used in traditional Chinese medicine and is a prized herbal folk medicine in various Asian countries [2]. The special pharmacological actions on gastritis, diabetes, cancer, fatigue, and ageing ranked *D. officinale* as “the first of the Chinese nine fairy herbs” [3,4,5]. The major bioactive compounds reported in *D. officinale* include alkaloids, polyphenols, and polysaccharides [2,6,7,8,9,10]. In particular, the key alkaloid, sesquiterpene dendrobine, has been reported as anti-hypertensive, anti-cancer, analgesic, and antipyretic [11,12].

Numerous studies reported detailed transcriptome profiles in various organs of *D. officinale* [10,13,14,15,16,17,18] and importantly, the biosynthetic pathways of alkaloids and polysaccharides were resolved. These genomic resources are essential tools for engineering *D*. *officinale* cultivars with enhanced levels of bioactive molecules. However, the transcriptome only represents the potential for a biological outcome [19]. In contrast, metabolomics is a more powerful technique because metabolites and their concentrations directly reflect the underlying biochemical activity and metabolic state of cells, tissues, or organisms. Therefore, integrating metabolomic and transcriptomic information will facilitate the elucidation of biosynthetic pathways of key bioactive compounds in *D. officinale*. Previously, Jin et al. [20] analyzed the metabolome of *D. officinale* stems but relatively few metabolites were reported in their studies, failing to provide an extensive picture of the metabolic richness in this species. Moreover, since different organs are used as the source material in herbal medicine and for the isolation of bioactive compounds [21], it is crucial to comprehensively examine the chemical composition of various organs in *D. officinale*. In the present work, we used the widely targeted metabolomic approach to detect and determine the concentration of hundreds of metabolites in the leaves and stems of *D. officinale*.

## 2. Results and Discussion

### 2.1. Qualitative Metabolic Profiling of D. officinale Leaves and Stems

**Figure 1:**
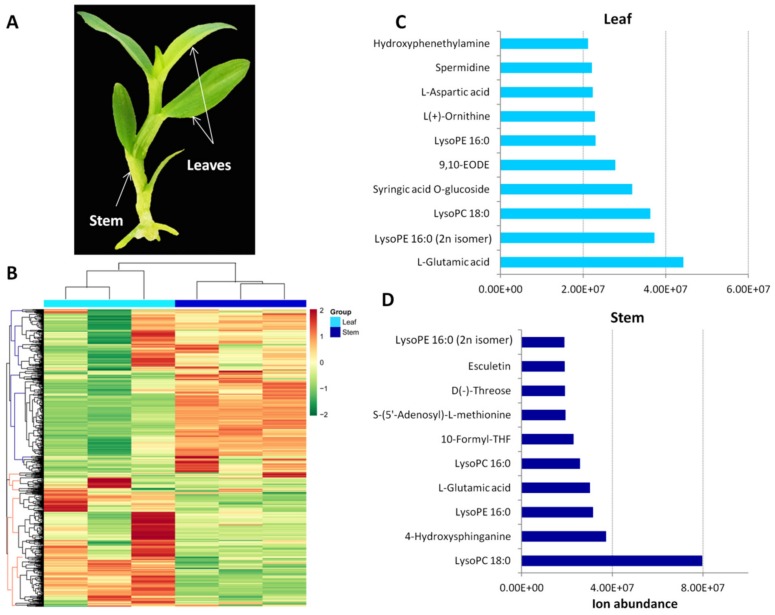
Metabolic profiling in leaf and stem samples of *Dendrobium officinale*. (**A**) A picture of *D. officinale* plant highlighting the two organs used for metabolomic analysis. (**B**) A heatmap hierarchical clustering of the detected metabolites in leaves and stems. The Log2 of the metabolite quantification was used. The columns correspond to the organs while the rows represent the different metabolites. (**C**) Top 10 most abundant metabolites detected in leaf samples. (**D**) Top 10 most abundant metabolites detected in stem samples.

In the present study, leaf and stem samples were used for metabolic profiling using the widely targeted metabolomics approach (Figure 1A). The self-compiled metabolite database MWDB of Metware Biotechnology Co., Ltd. (Wuhan, China) and the widely targeted metabolomics strategy offer a platform to detect a great diversity of metabolites in *D. officinale* as previously reported in tomatoes [22], *Prunus mira* [23], and hulless barley [24,25,26]. In total, 649 metabolites were successfully detected in both sample types (Table S1), which was approximately five times the number of metabolites detected in the previous study [20]. The diverse set of detected molecules could be roughly grouped into 32 major classes, predominantly organic acids, amino acids and their derivatives, nucleotides and their derivatives, and flavones (Table 1). In contrast, very few compounds belonging to the nicotinic acid derivatives, terpenoids, and proanthocyanidins classes were present in *D. officinale* leaves and stems. Alkaloids are the main desirable bioactive metabolites in *D. officinale* [12,27]. Here, we detected seven alkaloids, including hordenine, piperidine, quinine, isohemiphloin, betaine, theobromine, and trigonelline in both organs. Collectively, polyphenols were the major components of *D. officinale* metabolome with nine different classes of polyphenols (catechin derivatives, flavanones, flavones, flavone C-glycosides, flavonols, flavonolignans, hydroxycinnamoyl derivatives, isoflavones and anthocyanins) accounting for 1/3 of the total metabolites detected (Table 1). Apart from *D. officinale*, the genus *Dendrobium* contains over 40 species with high medicinal values [28]. The detailed metabolic profiles in the leaves and stems of *D. officinale* generated in the present study will facilitate cross-species comparison of key bioactive components [20,29,30]. Moreover, since most of the identified metabolites in this study have not yet been reported in *Dendrobium officinale* metabolic network, our work offers prospects for new bioactive compound discovery.

### 2.2. Quantitative Metabolic Profiling of D. officinale Leaves and Stems

The ion abundance of all detected metabolites is presented in Table S1. Based on the quantitative metabolic profiles of the two organs, heatmap hierarchical clustering could clearly distinguished leaf samples from stem samples (column), suggesting that although both organs contain approximately the same metabolites, the concentration of the metabolites vary considerably among them (Figure 1B). From the heatmap, two main groups of metabolites were obtained (lines); some metabolites were more highly accumulated in the stems than in the leaves and vice versa. Stems of *D. officinale* also known as “Fengdou” is the main organ exploited in traditional Chinese medicine [15]. However, it has been proposed that the leaves of *D. officinale* could be regarded as a new source of bioactive compounds [21] since fresh leaves account for approximately half of the total biomass [31] and contain more amino acids than stems [32]. The top 10 most abundant metabolites found in each organ are presented in Figure 1C,D and represent several classes of metabolites. The significant variation in metabolite concentration between leaves and stems of *D. officinale* uncovered in the present study showed that leaves contain key metabolites that may be of value as medicinal products.

There are ~200,000 metabolites in the plant kingdom and several metabolites such as aspirin, taxol, morphine, etc. are well characterized and widely used as drugs [33]. Continuous efforts to quantitatively uncover the spectrum of the primary and secondary metabolites in various plant species, in particular medicinal plants such as *D. officinale*, will provide prospects for finding not only novel natural sources for these well-characterized molecules but also new chemical entities for drug discovery and development [34]. The difficulty of identifying and isolating novel active compounds is the major hurdle in the traditional natural products discovery approach [35]. Therefore, efforts are ongoing to develop novel strategies aiming at shifting from the previous ‘grind and find’ model to a targeted discovery model, which requires information on the diverse metabolites present in the plant extracts [36,37]. In line with this, the metabolic data reported in this study combined with bioassay analysis targeting the enriched compounds in *D. officinale* organs may accelerate the discovery of bioactive compounds.

### 2.3. Differentially Accumulated Metabolites between Leaves and Stems in D. officinale

**Figure 2:**
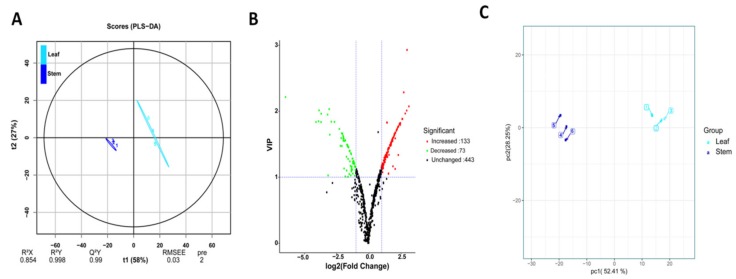
Identification of metabolites differentially accumulated in stems and leaves of D. nobile. (**A**) The partial least squares-discriminant analysis (PLS-DA) score plot. (**B**) Volcano plot showing the increased and decreased metabolites between leaves and stems. (**C**) Principal component analysis score plot based on the 206 differential metabolites.

We compared the quantitative metabolic profiles between stems and leaves in order to identify the compounds that differentially accumulated in each organ. Partial least squares-discriminant analysis (PLS-DA) is widely employed as an effective method for screening differential metabolites between groups [38,39,40]. We therefore applied the PLS-DA model to evaluate the difference in metabolite content between leaf and stem samples of *D. officinale*. The established PLS-DA model showed good fitness (R^2^X = 0.854, R^2^Y = 0.998) and predictability (Q^2^ = 0.99) (Figure 2A). The PLS-DA score plot shows a clear separation between leaf and stem samples with no points overlapped, illustrating the remarkable difference between both organs with respect to their metabolic content. The significant metabolites were selected based on the variable importance in projection (VIP) ≥1 and fold change ≥2 or fold change ≤0.5 [23]. In total, 206 compounds accumulated differentially between the two organs, with 73 metabolites preferentially accumulating in leaves and 133 compounds being significantly more abundant in stems (Figure 2B; Table S2). To confirm our result, we performed principle component analysis (PCA) to assess the overall clustering pattern of the two sample types based on the 206 differential metabolites (Figure 2C). The results show that the first two principal components, PC1 and PC2, could explain 52.41% and 28.25%, respectively, representing 80.66% of the total metabolic variation. PC1 clearly distinguished the two sample types, revealing that the content of the selected metabolites is significantly altered between leaves and stems of *D. officinale*. Accumulation of a large number of metabolites to high concentrations in the stems, particularly organic acids, amino acids and their derivatives, and nucleotides and their derivatives may explain why the stem is the preferred organ used as an herbal medicine [41,42]. We found that most of the significantly accumulated metabolites in the leaves are phenolic compounds (Table 2) which agreed with the previous report claiming that leaves of *D. officinale* could be regarded as a new antioxidant source [21]. In addition, the leaves contain many more lipids than the stems. Except for piperidine which was higher in the stems, most of the detected alkaloids had similar concentrations in both organs.

## 3. Materials and Methods

### 3.1. Plant Material

*Dendrobium officinale* Kimura et Migo plants were grown in a greenhouse (temperature = 24/18 °C day/night, relative humidity = 60% with natural light) at the Yunan Academy of Agricultural Sciences, Flower Research Institute, Yunan, China. Six-month-old plants were cultured in three replicated pots containing sandy soil with 10% of added compound fertilizer. Leaf and stem samples were collected from three independent plants, immediately frozen in liquid nitrogen and stored at −80°C until use for metabolomics analysis.

### 3.2. Metabolic Profiling

The preparation of samples, extract analysis, metabolite identification and quantification were performed at Wuhan MetWare Biotechnology Co., Ltd. (www.metware.cn) following their standard procedures and previously fully described by Zhang et al. [23].

#### 3.2.1. Sample Preparation and Extraction

The frozen samples were crushed using a mixer mill (MM 400, Retsch) with a zirconia bead for 1.5 min at 30 Hz. About 100 mg of powder was weighted and extracted overnight at 4 °C with 1 mL 70% aqueous methanol. Following centrifugation at 10,000× *g* for 10 min, the extracts were absorbed (CNWBOND Carbon-GCB SPE Cartridge, 250 mg, 3 mL; ANPEL, Shanghai, China, www.anpel.com.cn/cnw) and filtrated (SCAA-104, 0.22 μm pore size; ANPEL, Shanghai, China, http://www.anpel.com.cn/) before LC-MS analysis [43].

#### 3.2.2. HPLC Conditions

The sample extracts were analyzed using an LC-ESI-MS/MS system (HPLC, Shim-pack UFLC SHIMADZU CBM30A system, www.shimadzu.com.cn/; MS, Applied Biosystems 6500 Q TRAP, www.appliedbiosystems.com.cn/). The analytical conditions were as follows: HPLC column, Waters ACQUITY UPLC HSS T3 C18 (1.8 µm, 2.1 mm × 100 mm); solvent system, water (0.04% acetic acid): acetonitrile (0.04% acetic acid); gradient program, 100:0 *V*/*V* at 0 min, 5:95 *V*/*V* at 11 min, 5:95 *V*/*V* at 12 min, 95:5 *V*/*V* at 12.1 min, 95:5 *V*/*V* at 15 min; flow rate, 0.40 mL/min; temperature, 40 °C; injection volume: 2 μL. The effluent was alternatively connected to an ESI-triple quadrupole-linear ion trap (Q TRAP)-MS.

#### 3.2.3. ESI-Q TRAP-MS/MS

Linear ion trap (LIT) and triple quadrupole (QQQ) scans were acquired on a triple quadrupole-linear ion trap mass spectrometer (Q TRAP), API 6500 Q TRAP LC/MS/MS System, equipped with an ESI Turbo Ion-Spray interface, operating in a positive ion mode and controlled by Analyst 1.6 software (AB Sciex). The ESI source operation parameters were as follows: ion source, turbo spray; source temperature 500 °C; ion spray voltage (IS) 5500 V; ion source gas I (GSI), gas II (GSII), curtain gas (CUR) were set at 55, 60, and 25 psi, respectively; the collision gas (CAD) was high. Instrument tuning and mass calibration were performed with 10 and 100 μmol/L polypropylene glycol solutions in QQQ and LIT modes, respectively. Based on the self-built MetWare Database (http://www.metware.cn/) and metabolite information in the public database, the materials were qualitatively analyzed according to the secondary spectrum information and the isotope signal was removed during the analysis. QQQ scans were acquired as multiple reaction monitoring (MRM) experiments with collision gas (nitrogen) set to 5 psi [44]. De-clustering potential (DP) and collision energy (CE) for individual MRM transitions were done with further DP and CE optimization [43]. A specific set of MRM transitions were monitored for each period according to the metabolites eluted within this period.

### 3.3. Metabolite Data Analysis

Before the data analysis, quality control (QC) analysis was conducted to confirm the reliability of the data. The QC sample was prepared by the mixture of sample extracts and inserted into every two samples to monitor the changes in repeated analyses. Data matrices with the intensity of the metabolite features from the six samples were uploaded to the Analyst 1.6.1 software (AB SCIEX, Ontario, ON, Canada) for statistical analyses. The supervised multivariate method, PLS-DA, was used to resolve the metabolome differences between the two organs. The relative importance of each metabolite to the PLS-DA model was checked using the parameter called variable importance in projection (VIP). Metabolites with VIP ≥1 and fold change ≥2 or fold change ≤0.5 were considered as differential metabolites for group discrimination [23]. A heatmap based on the hierarchical cluster analysis method was performed in the R software (www.r-project.org).

## 4. Conclusions

In summary, we revealed hundreds of metabolites and their concentration in stem and leaf samples from the medicinal plant *D. officinale* for the first time. Although both organs contain similar metabolites, the metabolic concentrations were clearly different. The concentrations of many metabolites, particularly organic acids, amino acids and their derivatives, and nucleotides and their derivatives were higher in the stems than in the leaves. However, several polyphenol and lipid compounds were more enriched in the leaves. Our metabolic data suggest that the leaves of *D. officinale* could also be exploited as herbal medicine. Obtaining an extensive metabolite profile in plants has become a standard practice and represents the starting point for in-depth functional analysis of distinctly enriched compounds [34]. With the increasing genomic resources available in *D. officinale*, we propose that the reported metabolomic data from this study could be combined with other omics data to facilitate the elucidation of the biosynthetic pathway of important bioactive compounds. For example, our metabolite data could be integrated with available transcriptome data to infer gene-metabolic network, which is recognized nowadays as a powerful tool for biotechnology [45,46]. With the knowledge of the metabolic richness in *D. officinale*, future studies could, at a reduced cost, selectively target and investigate the natural variation and genetic architecture of key classes of metabolites such as alkaloids or polyphenols, which are receiving a lot of research attention due to the broad spectrum of their health-promoting effects [47]. Moreover, the metabolic data reported here combined with bioactivity screening may facilitate the discovery of new bioactive natural products by applying novel targeted and hypothesis-driven discovery models [36,37].

## Figures and Tables

**Table 1:** Classification of the 649 detected metabolites in *D. officinale* leaves and stems.

Class	Number of Compounds	Class	Number of Compounds
Organic Acids	72	Benzoic acid derivatives	15
Amino Acid Derivatives	65	Quinates and their derivatives	14
Nucleotides and Their Derivates	56	Coumarins	12
Flavones	46	Indole derivatives	11
Hydroxycinnamoyl Derivatives	37	Catechin derivatives	8
Lipids_Glycerophospholipids	33	Alcohols and polyols	7
Amino Acids	30	Alkaloids	7
Flavone C-Glycosides	30	Anthocyanins	7
Others	30	Cholines	7
Flavonols	24	Isoflavones	7
Lipids_Fatty Acids	22	Tryptamine derivatives	7
Carbohydrates	20	Nicotinic acid derivatives	4
Phenolamides	19	Pyridine derivatives	3
Lipids_Glycerolipids	18	Flavonolignans	2
Vitamins	17	Terpenoids	2
Flavanones	16	Proanthocyanidins	1

**Table 2:** Top 10 up and down accumulated metabolites in the stems as compared to the leaves.

Compounds	Class	Leaves (Ion Abundance)	Stems (Ion Abundance)	Fold Change (FC)	Log2 FC	Up/Down
Dl-2-Aminooctanoic Acid	Organic acids	6.17 × 10^4^	5.42 × 10^5^	8.77	3.13	up
Cyanidin O-malonyl-malonylhexoside	Anthocyanins	3.77 × 10^3^	2.99 × 10^4^	7.93	2.99	up
Methylglutaric Acid	Organic acids	8.88 × 10^3^	6.93 × 10^4^	7.80	2.96	up
2-Hydroxy-2-methyl butyric Acid	Organic acids	2.48 × 10^4^	1.79 × 10^5^	7.20	2.85	up
5-Aminolevulinate	Organic acids	9.40 × 10^5^	6.25 × 10^6^	6.66	2.73	up
Phenylacetyl-l-glutamine	Amino acid derivatives	2.54 × 10^4^	1.57 × 10^5^	6.20	2.63	up
4-Aminoindole	Indole derivatives	1.34 × 10^5^	8.24 × 10^5^	6.13	2.62	up
l-Glutamine	Amino acids	1.20 × 10^6^	7.34 × 10^6^	6.12	2.61	up
8-Methyl-2-oxo-4-phenyl-2H-chromen-7-yl 4-(hexyloxy)benzoate	Benzoic acid derivatives	2.58 × 10^6^	1.43 × 10^7^	5.55	2.47	up
5-methoxyindole-3-carbaldehyde	Indole derivatives	2.92 × 10^4^	1.61 × 10^5^	5.52	2.46	up
Biochanin A	Isoflavones	5.63 × 10^4^	6.97 × 10^3^	1.24 × 10^−1^	−3.01	down
Spermidine	Phenolamides	2.20 × 10^7^	2.65 × 10^6^	1.20 × 10^−1^	−3.06	down
Neochlorogenic acid (5-O-Caffeoylquinic acid)	Quinates and their derivatives	2.40 × 10^5^	2.71 × 10^4^	1.13 × 10^−1^	−3.14	down
MGMG (18:2) isomer2	Lipids_Glycerolipids	2.82 × 10^5^	3.11 × 10^4^	1.10 × 10^−1^	−3.18	down
Naringenin chalcone	Flavanones	5.53 × 10^5^	4.31 × 10^4^	7.80 × 10^−2^	−3.68	down
l-Asparagine	Amino acids	1.96 × 10^7^	1.52 × 10^6^	7.74 × 10^−2^	−3.69	down
Butin	Flavones	5.64 × 10^5^	3.93 × 10^4^	6.97 × 10^−2^	−3.84	down
l(+)-Ornithine	Amino acids	2.28 × 10^7^	1.54 × 10^6^	6.75 × 10^−2^	−3.89	down
Eriodictyol O-malonylhexoside	Flavanones	1.55 × 10^5^	9.13 × 10^3^	5.90 × 10^−2^	−4.08	down
2’-Deoxyinosine-5’-monophosphate	Nucleotides and their derivates	1.11 × 10^7^	1.26 × 10^5^	1.14 × 10^−2^	−6.45	down

## Supplementary Materials

The following are available online at https://www.mdpi.com/2218-1989/9/10/215/s1, Table S1. List of the metabolites detected in the leaf and stem samples from *Dendrobium officinale* and their ion abundance. Metabolites were detected in triplicate samples. Mix represents the mixture of sample extracts, Table S2. List of the differentially accumulated metabolites between leaf and stem samples in *Dendrobium officinale*.
